# Pericardial synovial sarcoma: challenges in diagnosis and management

**DOI:** 10.12688/f1000research.3-15.v2

**Published:** 2014-03-04

**Authors:** Prajakta Phatak, Jagadish Khanagavi, Wilbert S. Aronow, Sonam Puri, Yasmin Yusuf, Carmelo Puccio

**Affiliations:** 1 Department of Medicine, Westchester Medical Center / New York Medical College, Valhalla, NY 10595, USA; 2 Division of Cardiology, Westchester Medical Center/ New York Medical College, Valhalla, NY 10595, USA; 3 Department of Medicine, University of Connecticut, Farmington, CT 06030, USA; 4 Department of Pathology, Westchester Medical Center / New York Medical College, Valhalla, NY 10595, USA; 5 Division of Oncology, Westchester Medical Center/ New York Medical College, Valhalla, NY 10595, USA

## Abstract

**Introduction**: Pericardial synovial sarcoma is an extremely rare tumor with poor prognosis. Timely diagnosis and aggressive multimodal management improves patient outcome. We present our experience of diagnosis and management of a young patient with monophasic synovial sarcoma arising from pericardium.

**Case**: A 27-year-old man presented with dyspnea and cough of three weeks duration. Examination revealed sinus tachycardia, distant heart sounds and elevated jugular venous pressure. Chest X-ray showed widened mediastinum. Transthoracic echocardiogram (TTE) noted large pericardial effusion with tamponade physiology. Therapeutic pericardiocentesis yielded hemorrhagic fluid. Computed tomography (CT) of the chest showed persistent pericardial effusion and a left anterior mediastinal mass. Left anterior thoracotomy, pericardial window and left anterior mediastinotomy were done, revealing a well-encapsulated gelatinous tumor originating from the pericardium. Histology and immunohistochemical profile showed the tumor to be a monophasic synovial sarcoma. Fluorescent in-situ hybridization (FISH) was positive for SS18 (SYT) gene rearrangement on chromosome 18q11, substantiating the diagnosis. Work-up for metastases was negative. Neo-adjuvant chemotherapy with high dose ifosfamide led to substantial reduction in the size of the tumor. The patient underwent surgical resection and external beam radiation therapy (EBRT) post surgery. He had symptom-free survival for 8 months prior to local recurrence. This was managed with left lung upper lobectomy and follow-up chemotherapy with docetaxel. The patient is currently stable with an acceptable functional status.

**Conclusion:** In patients with pericardial effusions of unknown etiology, multiple modalities of cardiac imaging must be employed if there is suspicion of a pericardial mass. CT and magnetic resonance imaging (MRI) are useful to evaluate for pericardial thickening or masses in addition to TTE. Treatment of synovial sarcoma is not well established. Surgery is the cornerstone of treatment. In non-resectable tumors, aggressive neo-adjuvant chemotherapy with ifosfamide followed by surgical resection and EBRT may lead to improved outcome.

## Introduction

Synovial sarcomas are malignant soft tissue tumors in extremities with a predilection for juxta-articular location and are predominantly diagnosed in young adults. Synovial sarcoma arising from the pericardium is an extremely rare tumor. Although termed as synovial sarcoma, these tumors have now been recognized to arise from mesenchymal tissue and show epithelial differentiation ^1^. To the authors’ knowledge, only 20 cases of synovial sarcomas originating from the pericardium have been described in the English literature to date ^2^. This type of tumor is known to have a poor prognosis with no clarity about the best approach in managing this disease.

This case report describes a young man who presented to us with a cough and dyspnea and was diagnosed with cardiac tamponade due to this rare tumor. Pericardial effusions and cardiac tamponade secondary to malignancy are mostly secondary to metastases ^3^. Primary synovial sarcoma leading to tamponade is very rare, and to the best of our knowledge, has been reported only twice prior to our report ^4, 5^. We delineate the systematic approach to diagnosis using imaging modalities, histopathology and cytogenetic analysis. Due to its site of origin and histology, it was essential to distinguish this tumor from other soft tissue tumors, and cytogenetic analysis was crucial with regard to this. Although surgery is the cornerstone of treatment of this tumor, we used multiple modalities of treatment such as neo-adjuvant chemotherapy using high-dose ifosfamide and external beam radiation therapy (EBRT). Our patient had a good initial response to therapy and had improved survival compared to previous patients ^4, 5^. We believe that this case report and the discussion about diagnosis, management and interval monitoring for recurrence will add to the current literature and help in managing such cases in the future.

## Case report

**Figure 1.**
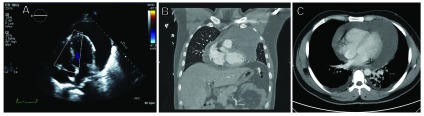
The transthoracic echocardiogram and CT chest findings. The echocardiogram image ( **a**) shows large pericardial effusion with collapse of the free wall of right atrium. The coronal ( **b**) and axial ( **c**) CT images show the effusion and an enormous heterogeneous mass in the pericardial cavity.

**Figure 2.**
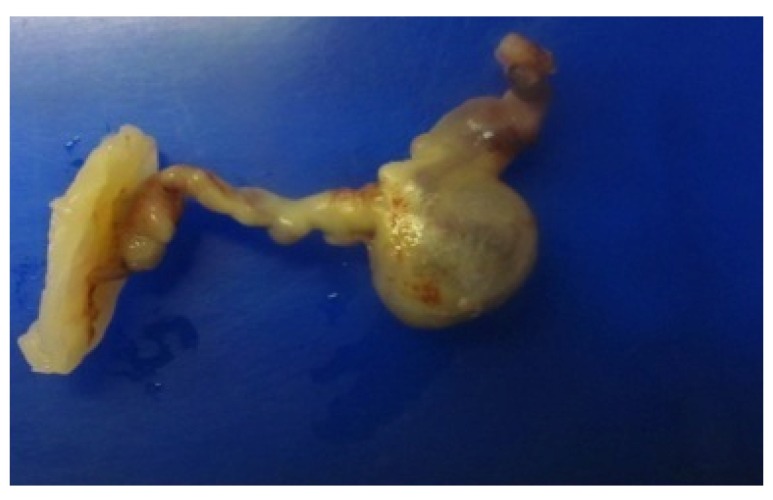
Gross Pathology. Left anterior thoracotomy, pericardial window and left anterior mediastinotomy were done, revealing a well-encapsulated gelatinous tumor originating from the pericardium (5.2 × 1.3 × 0.7 cm).

A 27-year-old Hispanic man with no significant prior medical history presented to the emergency room with shortness of breath and a cough for three weeks. He also complained of mild precordial chest pain and generalized fatigue. His family history was contained type 2 diabetes mellitus in both parents but reported no history of cancer or genetic diseases. His social history revealed that he worked as a gardener, was married with two kids, was a non-smoker, occasionally consumed alcohol and had no history of drug abuse. He gave no history of any psychological illnesses. A physical exam revealed sinus tachycardia, distant heart sounds and elevated jugular venous pressure and a normal blood pressure. Chest X-ray showed an impressive mediastinal enlargement. Transthoracic echocardiogram (TTE) showed a large pericardial effusion ( Figure 1a) and collapse of the right atrium during diastole along with respirophasic changes consistent with cardiac tamponade physiology. The patient underwent an emergent therapeutic pericardiocentesis that yielded hemorrhagic fluid. Cytological analysis of the fluid revealed no malignant cells. Computed tomography (CT) of the chest showed persistent pericardial effusion and a left anterior mediastinal mass ( Figures 1b and 1c). Work-up for possible metastases performed using Gallium scintigraphy showed no evidence of involvement of any other organs. Left anterior thoracotomy, pericardial window and left anterior mediastinotomy were done, revealing a well-encapsulated gelatinous tumor originating from the pericardium. The tumor grew from the inner surface of the pericardium near the origin of the superior vena cava and ascending aorta ( Figure 2). A portion of the pericardium where the tumor was attached was resected and the tumor was biopsied. Complete resection was not attempted due to the proximity and attachment to the great vessels.

**Figure 3.**
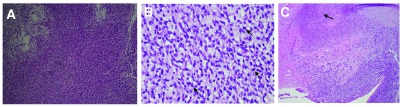
Biopsy specimen with routine hematoxylin-eosin stain. ( **3a**) (× 100 magnification) shows cellular monophasic tumor with spindle cells in fascicular growth pattern. ( **3b**) (× 400 magnification) shows cells in a myxoid background with many mitosis (arrows). ( **3c**) (× 100 magnification) shows large areas of necrosis (arrow).

**Figure 4.**
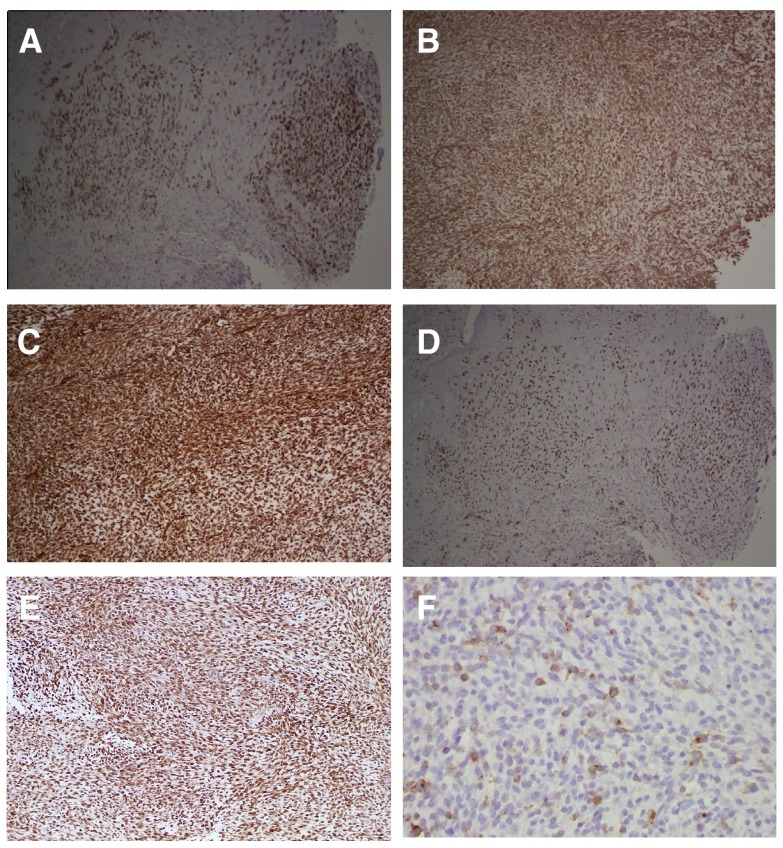
Immunohistochemistry. Tumor cells were strongly positive for: BCL-2 (× 100 magnification) ( **4a**), CD 99 (× 100 magnification) ( **4b**), Vimentin (× 100 magnification) ( **4c**), FLI1 (× 100 magnification) ( **4d**) and NSE (× 100 magnification) ( **4e**). They also showed focal scattered positivity for EMA (× 400 magnification) ( **4f**).

Review of pathology on routine hematoxylin-eosin stain showed that the tumor was monophasic and was composed of numerous spindle to oval cells in a fascicular growth pattern. The cells showed scant cytoplasm, oval to spindle nuclei with granular chromatin and with inconspicuous nucleoli ( Figure 3a). The tumor was mitotically active and showed 3–4 mitosis per high power field ( Figure 3b). Some areas showed myxoid background ( Figure 3b) and others showed large areas of necrosis ( Figure 3c). Immunohistochemistry showed that the tumor cells were strongly positive for BCL-2 ( Figure 4a), CD 99 ( Figure 4b), Vimentin ( Figure 4c), FLI1 ( Figure 4d) and neuron-specific enolase (NSE) ( Figure 4e). Stain for epithelial membrane antigen (EMA) showed focal scattered positive cells ( Figure 4f). Muscle markers (smooth muscle actin, desmin and myogenin), neural and melanoma marker (S-100), epithelial markers (AE1/AE3), CD57, CK5/6 and p63 were negative. Based on morphology and immunostaining results, a diagnosis of monophasic synovial sarcoma was made. Fluorescent *in-situ* hybridization (FISH) was done to confirm the diagnosis and to exclude Ewing sarcoma. It showed the presence of SS18 (SYT) gene rearrangement on chromosome 18q11, substantiating the diagnosis. Also the FISH assay for rearrangement or dosage abnormalities of the EWSR1 oncogene at Ewing sarcoma locus 22q12 was negative.

The patent was treated neoadjuvantly with high dose ifosfamide (2000 mg/m ^2^/day intravenous over 3 hours for 4 days) and mesna (1200 mg/m ^2^) repeated every 21 days between July and December 2012. On repeat CT imaging prior to planned surgical excision, the tumor had substantially decreased in size (> 50%) and the patient was noted to have elevation of left hemi-diaphragm indicating likely involvement of the left phrenic nerve.

A median sternotomy was performed and access to the heart and great vessels was obtained. The pericardium was opened and the tumor was seen abutting the bifurcation of the pulmonary artery and distal aortic arch, and involving a portion of the pericardium including the left phrenic nerve. The portion of the mass that was attached to the distal part of the pulmonary trunk and a lateral portion of the pericardium were resected. The tumor appeared to encase the left phrenic nerve consistent with the finding of elevation of left hemi-diaphragm on CT scan. This nerve was transected and its edges were controlled with hemoclips. The hemoclips also served as markers for possible future radiation treatment. The entire outline of aortic arch and the pulmonary artery were visible after removal of the thymus and the tumor, with no evidence of direct invasion. Once the tumor was mobilized laterally towards the left lung, it showed possible involvement of the wedge of the left upper lobe. The lung parenchyma was divided and a portion of the lung was transected from the left upper lobe. The entire tumor was removed en bloc with the surrounding tissue. The level 5 (subaortic) lymph node was also resected and sent for pathology.

The resected mediastinal mass (6.5 × 4 × 3 cm) was confirmed to be a monophasic synovial sarcoma on histology and immunohistochemistry. The adjacent lung was not involved by sarcoma. The three lymph nodes resected along with the mass were benign with non-necrotizing granulomas. The level 5 lymph node also showed non-necrotizing granulomas and was negative for malignancy. One of the resection margins was positive for malignancy. The patient recovered well after the repeat thoracotomy and EBRT was initiated 1-month post surgery. He had no recurrence of tumor on CT imaging at the first and second three-month intervals post surgery. Chest CT repeated at 9 months (third three-month interval) showed local recurrence. The patient was noted to have an anterior mediastinal mass extending to the left upper lobe of the lung and a left pleural effusion. There was no evidence for any distant metastases. The patient underwent resection of the mass and left upper lobectomy, left video-assisted thoracoscopy, drainage of pleural effusion and total decortication of left lung. Pathology confirmed the recurrence but the resection margins were free of malignancy. The pleural fluid was also negative for malignant cells. Chemotherapy was reinitiated with docetaxel (100mg/m ^2^) every 21 days along with steroid premedication with dexamethasone (4mg) for 5 days prior to treatment. This resulted in minor improvement on repeat CT imaging, but more importantly there was no further symptomatic worsening. The patient is clinically stable 18 months after diagnosis.

## Discussion

Primary cardiac tumors are rare, and large autopsy series have shown the prevalence of primary cardiac tumors to be approximately 0.02% ^6^. Soft tissue sarcomas account for 37% of these primary cardiac tumors ^7^. In descending order of frequency, these are angiosarcoma, fibrosarcoma, rhabdomyosarcoma, and malignant fibrous histiocytoma ^7, 8^. Synovial sarcomas of cardiac origin are rare, and those originating from the pericardium are exceedingly rare. The real incidence of pericardial synovial sarcoma may be underestimated though, as the precise site of origin of such tumors can be difficult to determine. To date, to the authors’ knowledge, only 20 cases have been reported in the English literature ^2^.

Synovial sarcomas are malignant soft tissue tumors with an aggressive growth pattern. Although they are predominantly diagnosed in extremities with a predilection for juxta-articular location, in young adults, they have been reported to occur in almost any organ ^9– 12^. They are classified as biphasic, monophasic and poorly differentiated based on their histology. Among the pericardial synovial sarcomas, the biphasic type (composed of an epithelial component and a spindle-cell component) appears to be more common than the monophasic type (only spindle-cells) and the poorly differentiated type (small round cells) ^2, 9– 12^. Our patient had a monophasic variant with predominant spindle to oval cells in a fascicular pattern. Some areas showed myxoid background as well as large areas of necrosis and the tumor was mitotically active.

Various other mediastinal and pericardial tumors (lymphoma, mesothelioma, thymoma, metastatic carcinomas, other soft tissue sarcomas) can have a similar appearance to synovial sarcoma on histology. Further analysis using immunohistochemistry is commonly done to differentiate between them. The specimens of our patient were uniformly positive for Vimentin, BCL-2 and CD99 (markers which are typical for synovial sarcoma) and showed focal positivity for EMA. We also noted absence of immunostain positivity for muscle markers (smooth muscle actin, desmin and myogenin), neural and melanoma marker (S-100), pankeratin (AE1/AE3), cytokeratin (CK5/6), CD57 and p63. This helped in arriving at a diagnosis of synovial sarcoma and to exclude other possible soft tissue tumors. In our patient, the tumor cells also stained positive for NSE and FLI1. FLI1 is a fusion gene that is usually positive in Ewing sarcoma, but some cases of synovial sarcoma also show FLI1 positivity ^13^.

Despite arriving at a diagnosis based on histology and immunohistochemistry, in order to further confirm diagnosis, cytogenetic analysis was done in our patient. Cytogenetic analysis is increasingly being used, especially in diagnosing those tumors arising in unusual sites. Confirming the histological diagnosis is crucial in determining therapy. More than 90% of synovial sarcomas demonstrate the presence of a reciprocal translocation t (X; 18) (p11.2; q11.2), and detection of this SSX-SYT fusion gene confirms the diagnosis ^1, 14– 17^. Reverse transcription-polymerase chain reaction (RT-PCR) or FISH can both be used to detect this gene. FISH has a higher sensitivity for detection and a better success rate than RT-PCR ^17^. In our patient, FISH assay was performed, and it showed the presence of SS18 (SYT) gene rearrangement on chromosome 18q11. Also, in our patient in view of the immunostain positivity for markers of Ewing sarcoma, the FISH assay was conducted for rearrangement or dosage abnormalities of EWSR1 oncogene at Ewing sarcoma locus 22q12, and the result was negative. This helped us to further confirm the diagnosis of synovial sarcoma and exclude Ewing sarcoma ^13^.

Management of synovial sarcoma of pericardial origin is challenging and needs a multimodal approach. It is an aggressive tumor with a poor prognosis. Most patients have a short survival time ^1, 2^. One exception is the case report of a patient whose survival exceeded 14 years and involved recurrences and multiple surgical resections ^4^. Surgical resection can lead to curative therapy. Most often, due to the site of origin, tumors are adherent to adjacent vital organs and are non-resectable at the time of diagnosis. Therefore, EBRT and less commonly neo-adjuvant chemotherapy are used to make the tumor resectable. In our patient, we could not attempt complete surgical resection. Therefore, after the initial biopsy and pericardial window, neo-adjuvant chemotherapy was done with six cycles of high dose ifosfamide (2000mg/m ^2^) and mesna. Due to the rarity of these tumors, it is very difficult to establish an optimal regimen for chemotherapy. In general synovial sarcomas are chemo-sensitive to ifosfamide and show a good response. A case series of 13 patients with synovial sarcoma treated with high-dose ifosfamide showed a response in all patients, with 4 clinical remissions achieved ^18^. Combining doxorubicin with ifosfamide may achieve a higher response rate (58%) ^19^ but patient tolerability and side effects need to be considered. The initial favorable response to neoadjuvant high dose ifosfamide in our patient made the tumor resectable and may be a viable alternative to EBRT alone in non-resectable tumors. Gu *et al.* have reported using the collagen gel droplet embedded culture-drug sensitivity test (CD-DST) prior to determining the therapeutic value of chemotherapy in their patient with synovial sarcoma ^20^. It may be useful to have CD-DST as a screening method for the selection of effective anticancer drug regimen for each individual patient ^2^.

EBRT is a very useful modality of treatment post surgery when the margins of resection are positive. In our patient, EBRT was initiated one month after surgery. Previous reports have shown similar favorable results ^21^. To the authors’ knowledge only EBRT has been employed in previous cases. Intraoperative radiation therapy (IORT) with a radiation dose of 15–45 Gray appeared to be useful for local control and to be more effective in patients without distant metastases at the first visit in a study that included 24 patients with soft tissue sarcomas (1 case of synovial sarcoma) ^22^. But, lack of institutional experience with IORT dissuaded us from using it in our patient. Close monitoring for recurrence is very crucial in management of this tumor. CT or magnetic resonance imaging should be performed every 6 months to detect subclinical recurrences ^4^. We performed CT imaging every three months along with regular clinical follow-up. Repeat imaging is essential to be able to diagnose subclinical recurrence and initiate repeat broad surgical resection and follow-up chemotherapy if needed. We used the anti-mitotic agent docetaxel as well as left lung upper lobe resection for treating the recurrence. Adjuvant therapy with single agent docetaxel is not considered standard therapy for synovial sarcoma, and adjuvant therapy after resection of metastases is also controversial. Our patient was treated with docetaxel for his local recurrence based on evidence showing that docetaxel is an efficacious and tolerable treatment as rescue medication in ifosfamide-resistant locally advanced or metastatic soft tissue sarcoma ^23^.

## Conclusions

This case report describes a case of monophasic synovial sarcoma of pericardial origin presenting as pericardial effusion with cardiac tamponade physiology in a young man. It is an extremely rare tumor, and systematic approach to diagnosis and management are crucial. We emphasize the use of cytogenetic analysis in confirming the diagnosis and excluding tumors that might mimic synovial sarcoma on immunohistochemistry. This case also highlights the use of neo-adjuvant chemotherapy using high dose ifosfamide as part of multimodal approach to treatment.

## Consent

Written informed consent for publication of clinical details and clinical images was obtained from the patient.
