# Supplementary material for: Pericardial synovial sarcoma: challenges in diagnosis and management
Source: F1000Res. 2014 Mar 4;3:15. Originally published 2014 Jan 17. [Version 2] doi: 10.12688/f1000research.3-15.v2 (PMC3954165; doi:10.12688/f1000research.3-15.v2)
Supplement: Peer review history

# Peer review: Referee response for version 2

**Published:** 2014-03-06

**Authors:** Nikhil Mukhi

**Affiliations:** 1 Division of Oncology, SUNY Downstate Medical Center, Brooklyn, NY, USA

The changes are appreciated and the format is appropriate.

I have read this submission. I believe that I have an appropriate level of expertise to confirm that it is of an acceptable scientific standard.

# Peer review: Referee response for version 2

**Published:** 2014-03-04

**Authors:** Edwin Choy

**Affiliations:** 1 Dana-Farber/Harvard Cancer Center, Harvard University, Boston, MA, USA

The changes made were satisfactory.

I have read this submission. I believe that I have an appropriate level of expertise to confirm that it is of an acceptable scientific standard.

# Peer review: Referee response for version 1

**Published:** 2014-02-24

**Authors:** Gurpreet S Lamba

**Affiliations:** 1 Hematology Oncology Division, Department of Medicine, University of Vermont, Burlington, VT, USA

This is an interesting paper and is well written. It would definitely help future clinicians who might encounter this rare diagnosis. However I think that for the benefit of these clinicians the authors should include more details on the chemotherapy regimen, including the number of days ifosfamide was administered for and the frequency of the cycle. More details about the surgery and radiation would also be helpful.

I have read this submission. I believe that I have an appropriate level of expertise to confirm that it is of an acceptable scientific standard, however I have significant reservations, as outlined above.

# Peer review: Referee response for version 1

**Published:** 2014-02-12

**Authors:** Edwin Choy

**Affiliations:** 1 Dana-Farber/Harvard Cancer Center, Harvard University, Boston, MA, USA

In this case report, the authors offer a clinicopathologic description of a case of pericardial synovial sarcoma. 

 

There are several major points that need to be addressed:

1. The patient was treated neoadjuvantly with "high dose" ifosfamide, but the authors describe using 2000 mg/m2 per cycle, which is actually considered quite a low dose for treatment of sarcomas. This is either an error (perhaps 2000 mg/m2 was given each day for 3, 4, or 5 days per cycle?) or a very low dose was used intentionally. Regardless, the patient apparently benefitted with some tumor shrinkage, which allowed for surgical resection. 
   
2. A description of the surgery is lacking. We do not know how much pericardium was removed or if lymph nodes were sampled, as sarcomas are known to metastasize lymphatically as well as hematogenously. 
   
3. Although postoperative EBRT was administered, there is no discussion about whether intraoperative XRT or proton therapy was considered. 
   
4. Upon disease recurrence with a mediastinal mass and pleural effusion, a lobectomy and decortication was performed. We are not told if malignant cells were seen in the pleural fluid, in which case the entire pleura would be considered contaminated. 
   
5. Post-op, the patient was treated "adjuvantly" with docetaxel. Here, the authors need to be careful to point out that single agent docetaxel is not considered standard therapy for synovial sarcoma, and adjuvant therapy after a metastasectomy is also quite a controversial strategy. It would be wrong if readers interpreted this report as an endorsement of post-op single agent docetaxel after a metastasectomy. 

In summary, it is not clear what we learn from this case report except that the patient apparently sustained a partial tumor response to ifosfamide chemotherapy, tolerated an R1 or R2 surgery, and despite post-op radiation, sustained a regional/metastatic recurrence. A more thorough discussion about the complexities and controversies of clinical decision making in patients such as this would make such case reports more informative to the audience.

I have read this submission. I believe that I have an appropriate level of expertise to state that I do not consider it to be of an acceptable scientific standard, for reasons outlined above.

# Peer review: Referee response for version 1

**Published:** 2014-01-27

**Authors:** Nikhil Mukhi

**Affiliations:** 1 Division of Oncology, SUNY Downstate Medical Center, Brooklyn, NY, USA

This is a very well written article, and I think it makes a positive contribution to our current knowledge of cardiac sarcomas. The authors appropriately mention that these tumors are rare and no standard of care exists. However, this patient in the relapse setting was treated with single agent docetaxel. In phase II trials of soft tissue sarcomas, docetaxel has been used in combination with gemcitabine due to their synergistic effect ( Maki RG *et al.*, 2007). It is unclear why patient was only given docetaxel. 

It is however very interesting to see that patient responded well to therapy and is stable 18 months post treatment. This article may help guide therapy in these tumors.

I have read this submission. I believe that I have an appropriate level of expertise to confirm that it is of an acceptable scientific standard.

# Response

**Published:** 2014-01-29

**Authors:** Wilbert Aronow

**Affiliations:** Cardiology Division, New York Medical College, USA

We thank Dr. Mukhi very much for his review and approval of our case report. Our patient was treated with docetaxel for his local recurrence based on evidence showing that docetaxel is an efficacious and tolerable treatment as rescue medication in ifosfamide-resistant locally advanced or metastatic soft tissue sarcoma ( Kostler *et al.* 2001). Maki *et al.* (2007) showed that gemcitabine-docetaxel yielded a better response compared to gemcitabine alone when used for initial treatment rather than as a rescue treatment option. We are of the opinion that although our patient had a favourable response, it may be useful to have CD-DST as a method for the selection of an effective anticancer drug regimen for each individual patient for both primary treatment and to select the rescue chemotherapy.
